# Determinants of Kangaroo Mother Care Uptake for Small Babies Along the Health Facility to Community Continuum in Karnataka, India

**DOI:** 10.9745/GHSP-D-22-00457

**Published:** 2023-06-21

**Authors:** Maryann Washington, Leah Macaden, Annetta Smith, Sumithra Selvam, Prem K. Mony

**Affiliations:** a Division of Epidemiology and Population Health, St. Johns Research Institute, Bangalore, India.; b Nursing Studies, University of Edinburgh, Edinburgh, United Kingdom.; c Department of Nursing and Midwifery, University of the Highlands and Islands, Inverness, United Kingdom.

## Abstract

**Introduction:**

Kangaroo mother care (KMC) scale-up is a proposed strategy to accelerate reduction in neonatal mortality rates. We aimed to identify determinants of KMC uptake for small babies (less than 2,000 g birth weight) along the health facility to community continuum in Karnataka, India.

**Methods:**

From June 2017 to March 2020, data on characteristics of health facilities and health care workers (HCWs) from 8 purposively selected health facilities were assessed. Knowledge, attitude, and support the mothers received for KMC uptake were assessed once between 4 weeks and 8 weeks unadjusted age of the cohort of babies. Secondary data on KMC were obtained from the district-wide implementation research project database. Bivariate analysis was used to assess the association of characteristics of health facilities, HCWs, mothers, and small babies with the day of KMC initiation and its duration. Log-binomial regression analysis was then computed to identify determinants of KMC.

**Results:**

We recruited 227 (91.5%) of 248 babies eligible to participate with a mean unadjusted age of 35.6 days (±7.5) and 1,693.9 g (±263.1 g) birth weight. KMC was initiated for 95.2% of 227 babies at the health facility; initiated at 3 days or earlier of life for 59.6% of 226 babies; and babies continued to receive KMC for more than 4 weeks (30.2 days [±8.4]) at home. Determinants of KMC initiation were HCWs’ attitudes, initiation support at the health facility, and place of hospitalization. Determinants of KMC maintenance at the health facility were HCWs’ skills and support the mother received at the facility after initiating KMC. Place of hospitalization and HCWs’ knowledge determined KMC duration at home 1 week after discharge.

**Conclusion:**

These findings emphasize the importance of competent HCWs and support for mothers at the health facility for initiation and maintenance of KMC within the health facility and 1 week after discharge.

## Abstract (precis)

In Karnataka, India, the initiation and duration of kangaroo mother care (KMC) for small babies improved following KMC support for mothers and family members at the health facility and improved KMC competence of health care workers.

## Teaser

**Key Findings:**

- Place of hospitalization determines the early initiation and duration of kangaroo mother care (KMC).
- The competence of health care workers (HCWs) enables KMC initiation and optimal duration before discharge and at least until the seventh day after discharge.
- Support for mothers at the health facility to initiate and maintain KMC determines its early initiation and longer duration.

**Key Implications:**

- Health facility managers need to invest in contextual, supportive capacity-building strategies for HCWs and community health workers (CHWs) to facilitate the uptake of KMC along the health facility to community continuum.
- Health facility managers and implementers (HCWs and CHWs) need to find ways to support mothers along the health facility to community continuum to initiate and maintain KMC uptake for as long as required.

## INTRODUCTION

Globally, preterm babies and low birth weight (LBW) neonates are at the greatest risk for health problems, such as unstable body temperature, feeding difficulties, infections, low blood sugar, and breathing difficulties, all of which increase their risk of mortality.^1^ This burden of neonatal morbidity and mortality is borne by low- and middle-income countries (LMICs).^2^ Of all preterm births, 84% of babies born are moderate or late preterm^2^^,^^3^ and require only supportive and essential neonatal care (ENC) without intensive therapy for survival.^3^ Given the inequities that exist between high-income countries and LMICs in access to intensive care and high-tech environments with a trained workforce and the resultant 90:10 survival gap of preterm births less than 28 weeks of gestational age,^1^^,^^3^ LMICs need to explore different models to scale up ENC packages. Constraints with finance, infrastructure, and human resources in LMICs, including India, do not justify investments solely directed toward scale-up of neonatal intensive care units for reduction in neonatal mortality.^4^ Instead, a strategic balance of investments to scale up cost-effective ENC packages for LBW babies, especially stable small babies, would likely be more sustainable for LMICs to reach the Sustainable Development Goal 3 target of neonatal mortality rates less than 12 per 1,000 live births by 2030.^5^

Three evidence-based, cost-effective packages are recommended for scale-up globally toward accelerated reduction in neonatal mortality.^2^ The first, targeting all neonates irrespective of gestational age or birth weight, is ENC, which encompasses thermal care, hygienic cord and skin care, and early initiation of and exclusive breastfeeding. The second package, intended for approximately 5%–10% of neonates^2^^,^^6^ who do not breathe spontaneously at birth, includes basic neonatal resuscitation with a possibility of averting 30% of term neonatal deaths and 5%–10% of preterm deaths at the community and health facility.^7^^–^^10^ The third package recommended is kangaroo mother care (KMC) and breastfeeding support for mothers with LBW babies,^2^^,^^11^ shown to reduce mortality at discharge by 40%.^12^^,^^13^

Despite policy recommendations on KMC for stable small babies (<2,000 g) at birth,^14^ a report on KMC practice from only secondary public health facilities in states of India indicated only 0%–20% of LBW (<2,500 g) babies admitted to Level II neonatal care units (referred to as special newborn care units) from 12 of 20 states had received KMC.^15^ One could extrapolate that there is low coverage of LBW babies receiving KMC in these newborn units. KMC, a simple intervention that includes skin-to-skin contact (SSC) of optimal duration (8 hours or more) for 4–6 weeks of life, exclusive breastfeeding, early discharge, and close follow-up,^11^^,^^14^^,^^16^ is apparently not widely implemented. Universal coverage of all stable LBW babies with KMC is mandated if India is to achieve the target of 75% of all LBW babies covered with KMC by 2025 and 90% of them by 2030.^17^

Despite policy recommendations on KMC for stable small babies at birth, a report on KMC practice in 20 states of India indicated that only 0%–20% of LBW babies admitted to special newborn care units from 12 of the states had received KMC.

Research has shown a reduction of neonatal morbidity and mortality when babies with a birth weight of 1,000 g–1,799 g were provided with immediate and continuous KMC^18^ and when stable LBW babies were initiated early with KMC that was continued with optimal duration.^12^^,^^13^^,^^19^^,^^20^ Subsequently, several systematic reviews and meta-analyses^21^^–^^28^ reported on barriers and facilitators for KMC implementation from the perspectives of health systems, health care workers (HCWs), parents, and the community. Yet, none of these studies explored the direct association of the facilitators with KMC uptake.

A district-wide implementation research project in 3 Indian states recently demonstrated that coverage of more than 80% of small babies with KMC was possible with a comprehensive model comprising a package of the following interventions.^29^^,^^30^

1. Advocacy with the state and district leadership to reinforce the policy for KMC implementation^22^^,^^25^; ensure health facility preparedness for KMC implementation^22^^,^^25^; mobilize funds^23^ for infrastructure changes, equipment procurement, and support of health workforce^21^^,^^25^; establish a quality improvement committee; and monitor KMC coverage by documentation of its uptake.^22^^–^^23^
2. Capacity-building of HCWs through on-site mentorship and supportive supervision^21^^,^^22^^,^^28^^,^^29^ to identify eligible small babies (those who did not require advanced care for health problems and were able to directly breastfeed or be fed breastmilk through alternative modes) through accurate birth weight checking, implementing protocols, documenting care, including KMC, and facilitating their skills for initiation and maintenance of KMC.^29^^–^^32^
3. Community mobilization and capacity-building of CHWs for continuation of KMC at home through mentorship and support^21^; improving linkages of CHWs and HCWs^22^; and ensuring close follow-up of the small baby at home after discharge from the health facility.^29^^–^^31^ The CHWs’ capacities to solve problems and address barriers to facilitate increase of KMC duration at home was targeted.^29^^,^^31^ Additional components of the comprehensive model included identification of KMC champions (mothers with experience in providing KMC) in the community and conducting community events^29^^,^^31^ to promote KMC coverage.

Yet “what” within this comprehensive model directly influenced KMC uptake along the health facility to community continuum was unanswered. Both the plurality of the place of childbirth^33^ and the circumstance of early self-discharge by mothers after childbirth^34^^–^^35^ in India necessitated a strategic view on specific variables that managers and HCWs could focus on to increase KMC uptake along the health facility to community continuum.

**FIGURE 1:**
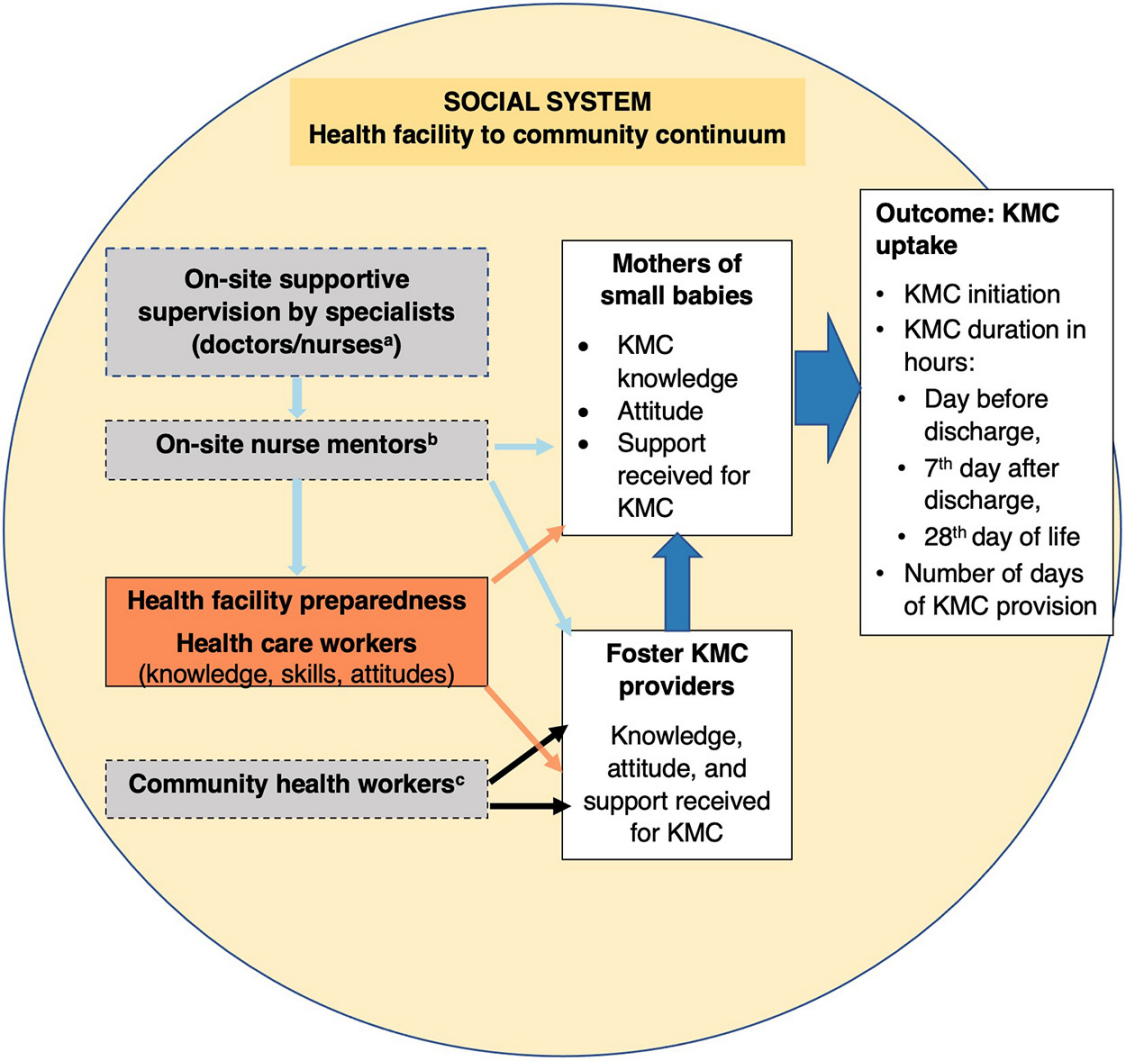
Conceptual Framework for KMC Uptake Study in Karnataka, India Abbreviation: KMC, kangaroo mother care. ^a^Supportive supervision specialists were not recruited because they only facilitated processes of health facility preparedness or in capacity-building of health care workers (knowledge, attitude, and skills). ^b^Not recruited to the study, but the role of on-site nurse mentors was indirectly studied through responses of mothers and foster KMC providers on support for KMC uptake at the health facility. ^c^Community health workers’ input was indirectly studied from responses of mothers and foster KMC providers on the KMC maintenance support at home, after discharge from the health facility.

KMC uptake by mothers along the health facility to community continuum was the targeted outcome behavior in our study. Behavior is known to be a function of intention (dependent on knowledge, attitudes, social factors, and belief of their roles); habitual responses (situation-behavior sequences that are automatic); and situational constraints and conditions (physical and social environment—health facility and at home).^36^ Thus, based on the literature reviewed on facilitators for KMC uptake,^21^^–^^28^ we developed a conceptual framework to guide the methodology of our study (Figure 1). The independent variables chosen were not part of the package of interventions within the comprehensive model of the district-wide project^29^ but were presumed as possible outputs of the package of interventions. For example, presumed outputs of capacity-building initiatives through on-site mentoring and specialist supportive supervision for HCWs were changes in their knowledge, attitude, and skills to implement KMC. These variables included: health facility preparedness^21^^,^^23^^,^^25^^,^^27^; HCWs’ knowledge, attitude, and skills on KMC^21^^,^^23^^,^^25^^,^^27^^,^^28^; KMC initiation and maintenance support received at the health facility^24^^,^^26^; mothers’ knowledge, attitude, and KMC maintenance support received at home^22^^–^^24^; and the babies’ sex, weight, and health status at birth.^21^^,^^28^ To the best of the authors’ knowledge, this study is the first to test the following hypotheses: (1) health facility preparedness increases early initiation and duration of KMC before discharge; (2) HCWs’ optimal knowledge, attitude, and skills are likely to impact uptake of KMC by mothers; and (3) mothers who are supported at the health facility by HCWs and at home by family members and CHWs are likely to practice KMC for longer duration at the health facility and subsequently at home, respectively.

## METHODS

### Design

The district-wide implementation research project was conducted in Karnataka to inform policymakers and health officials on a comprehensive model for KMC scale-up.^29^^,^^30^ Operational research with observation was used for our study to assess what within this comprehensive model influenced KMC uptake along the health facility to community continuum (Box). Operational research helps to find possible solutions to problems. Hence, we chose this approach to test the hypotheses and to inform health managers and HCWs what would determine uptake.

BOX Health Facility Manager’s Role to Improve KMC Uptake Along the Health Facility to Community Continuum^a^

**Support mechanisms to build competence of health care workers (HCWs) and community health workers (CHWs) through commitment of local medical and nursing institutions to facilitate:**

- Open visitation policy to enable key family members to begin their role as foster kangaroo mother care (KMC) providers and documentation of KMC to monitor progress.
- Health facility preparedness, including having a separate space, adjustable beds, feeding equipment, and amenities for mothers; education materials; and KMC champions to propagate KMC practice.
- Skill-based sensitization training on KMC implementation with emphasis on soft skills (e.g., communication, teamwork, and providing support).
- On-site mentoring at all public and private health facilities and in the community.
- Supportive supervision by specialists, including nurses/doctors.
- Monthly team meetings to reinforce linkages and reflect on progress of KMC implementation.

**Community mobilization for KMC through:**

- Provision of information/link cards to families to communicate to CHWs about their discharge.
- Dissemination of information on KMC by champions (i.e., mothers with experience in providing KMC) and using multiple methods (e.g., street plays, festival drama, local TV programs, posters and billboards and use of mHealth messaging).

^a^As indicated by the district-wide study reported on by Mony et al. and Jayanna et al.^29^^,^^30^

### Setting

Gangawati is 1 of 4 subdistricts—comprising 145 villages and an estimated population of 105,529^37^—that is situated east of district headquarters in Koppal, Karnataka. This subdistrict was chosen as the research site purposively because it was the second largest, most urbanized subdistrict and yet was reported to have the lowest male literacy rate (76.51%) among the 4 subdistricts of Koppal district. The Human Development Index (indicators include living standard, health index, and education index) of Gangawati is 0.594, ranking second among the 4 subdistricts of Koppal.^38^ It has 15 public health facilities—1 secondary level subdistrict hospital; 14 primary level health facilities, including 3 community health centers (CHCs) and 11 primary health centers (PHCs); and 18 private health facilities that include 6 level I or II neonatal care units and 12 childbirth/maternity centers.

### Population

The study population consisted of (1) all public and private health facilities with capabilities for providing ENC; (2) HCWs (nurses, doctors, health assistants, and counselors); (3) mothers and foster KMC (fKMC) providers (a healthy family member who provided SSC to the baby if the mother was unwell or while she had respite); and (4) small babies residing in the subdistrict.

### Sampling Technique and Size

Purposive sampling was used to select health facilities and all their available HCWs. Thus, 8 health facilities (7 public that included the subdistrict hospital, 6 primary health facilities [3 PHCs and 3 CHCs], and 1 private Level II neonatal care unit of the six private health facilities offering neonatal services) were selected because they provided access to 80% of small babies in the subdistrict and represented the different types of health facilities. All HCWs (n=79) available from the chosen health facilities were recruited based on the assumption that they would be representative of HCWs in the subdistrict.

Consecutive nonprobability sampling was adopted in our study to select small babies with rolling enrollment from an eligible population. The small babies’ sample size was calculated based on the assumption that 40% of babies would be provided with 8 hours or more per day of KMC by the end of the district-wide project, given that when the district-wide project began, coverage of babies had improved from less than 2% to 5% within 3 months (August to October 2016) in the district headquarters.^29^^,^^31^ Hence, with a relative precision of 15% and 95% confidence interval, 175 stable small babies were required. Small babies, irrespective of their mothers’ sociodemographic background, were recruited if they were born or hospitalized in any of the health facilities located in the subdistrict, had survived 4–8 weeks of unadjusted age, and were resident temporarily or permanently in the subdistrict. After accounting for 20% attrition, the revised sample size was calculated to be 210 babies. Mothers of babies recruited to the study and all available fKMC providers, with preference for males if there were more than 2 in a family, were automatically selected.

### Instruments

All instruments were investigator developed, field-tested, and validated. To assess health facility preparedness, an observation checklist was used that consisted of 10 items relevant to KMC implementation within the following domains: (1) health workforce that included availability of specialists and support staff and whether HCWs were trained on KMC; (2) health information systems that included availability of a case sheet to document KMC and KMC coverage reporting; (3) health service delivery that included availability of a separate area/unit for mothers to provide KMC with amenities of food, bathroom with water, adjustable beds, digital weighing machine, feeding equipment, and education materials on KMC; (4) leadership that included availability of a policy for KMC implementation.

A questionnaire was used to assess the knowledge of HCWs. This included 35 items organized under the following domains: identification of eligible babies for KMC; components and requirements for KMC; provision and monitoring of a baby on KMC; and maintenance of KMC (internal consistency to establish reliability was at 0.8). The attitude was assessed based on responses on a 0–4 Likert scale with 15 items organized under benefits of KMC, implementation of KMC, and KMC uptake (reliability of responses was well dispersed and established at 0.81). Skills of HCWs were assessed using an Objective Structured Clinical Assessment format with trained observers on 5 KMC-related skill stations. The skill stations included: (1) checking the weight and temperature and swaddling a small baby; (2) counseling a mother/family member for KMC uptake; (3) expressing breast milk and feeding a small baby using a cup or pallada (a cup-like utensil); (4) inserting an orogastric tube, calculating feed volume, and giving a tube feed; (5) counseling a mother/family with a small baby at discharge.

The knowledge, attitude, and support received by mothers and fKMC providers were assessed using a questionnaire. Responses of mothers were scored against a predetermined scoring key. Support was categorized as follows.

1. KMC initiation support, including counseling and assistance to initiate KMC at the health facility.
2. KMC maintenance support at the health facility, measured by the number of people to assist the mother to continue KMC, presence of a fKMC provider, and availability of a KMC kit (which consists of a bag and binder to position the baby securely for KMC, a cap and socks for the baby, and a pallada to feed the baby expressed breastmilk if needed).
3. KMC maintenance support at home, measured by the number of people who helped the mother with domestic chores or childcare at home; presence of and hours of KMC provided by fKMC provider; knowledge, attitude, and support score of fKMC provider; and support provided by the CHWs.

### Data Collection

**FIGURE 2:**
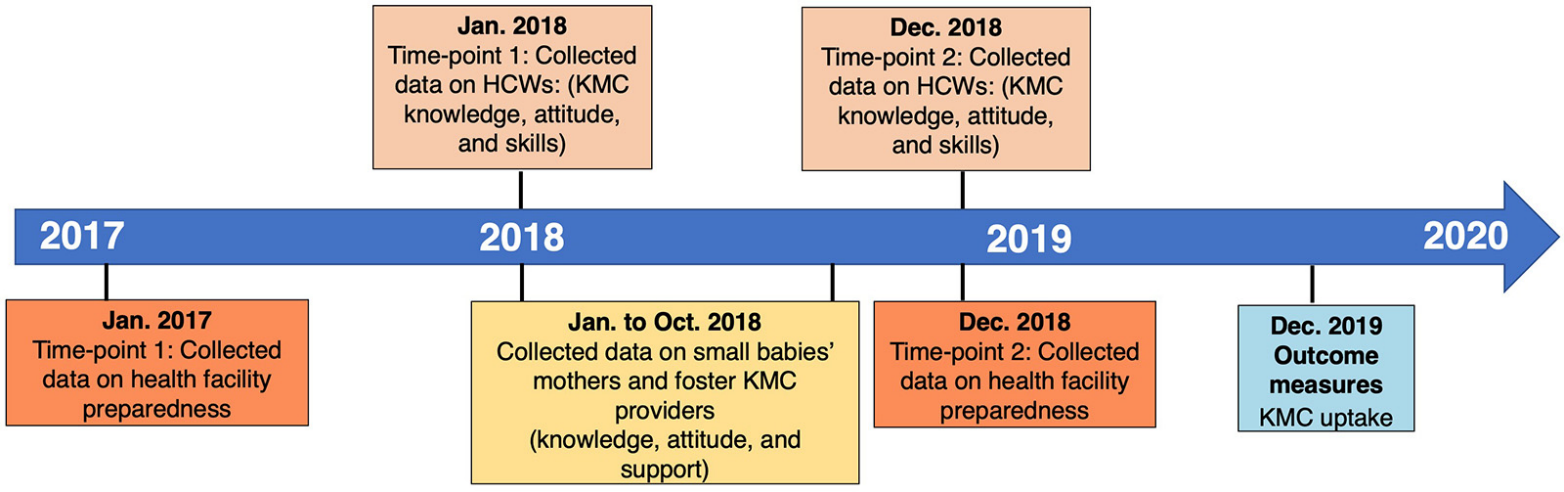
Data Collection Points for KMC Uptake Study in Karnataka, India, June 2017–March 2020 Abbreviations: HCW, health care worker; KMC, kangaroo mother care.

Data for this study were collected in a phased manner from June 2017 to March 2020 (Figure 2). Data on health facility preparedness were collected at 2 time points—once before the start of the district-wide project in this subdistrict (June 2017) and then at the end of the project (December 2018). Data on knowledge, attitude, and skills of HCWs on KMC implementation were also collected at 2 time points—once in January 2018 (6 months from the start of the district-wide project in this subdistrict) and then in December 2018. Every month, starting from January 2018, the cohort of small babies were identified from the district-wide project staff’s list of small babies who had completed 4 weeks of life (born or referred to any of the health facilities within the subdistrict). The project staff then recruited mothers of these babies; 1 week later, the investigator collected data on knowledge, attitude, and support received for KMC uptake in the mothers’ respective homes. Data from the available fKMC providers were also collected. Thus, data from mothers and fKMC providers were collected between January and October 2018 until the sample size was obtained.

Secondary data on KMC duration were collected on the day before discharge, seventh day after discharge from the health facility, and 28^th^ day of life after the district-wide project was completed. The investigator was blinded to the KMC details at the time of collecting information from mothers and fKMC providers. KMC data were retrieved from the evaluation team of the district-wide project for babies born in the period from December 2017 to January 2018 and from the district-wide project database for babies born in the period from February 2018 to September 2018.

### Data Analysis

**FIGURE 3:**
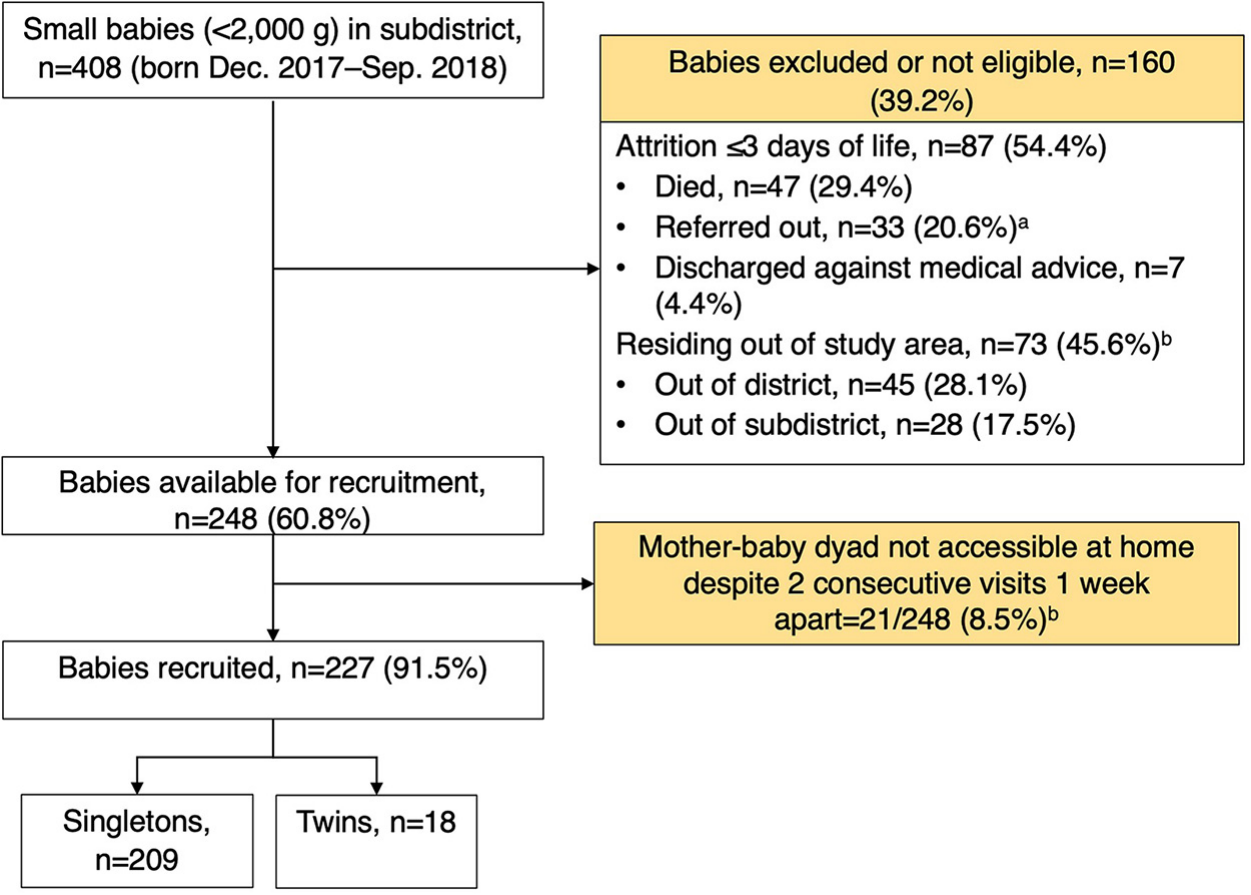
Participant Recruitment Process for KMC Uptake Study in Karnataka, India Abbreviation: KMC, kangaroo mother care. ^a^3 babies did not survive 28 days of life. ^b^1 baby did not survive 28 days of life.

Although initially reported as 141 babies excluded,^39^ in this study, we found 160 (39.2%) of the 408 small babies born between December 2017 and September 2018 were not eligible for recruitment, as per criteria of attrition in the district-wide project (Figure 3).^29^ Thus, 227 (91.5%) of 248 babies with a mean unadjusted age of 35.6 days (±7.5) and 1,693.9 g (±263.1) birth weight were recruited to this study.

The independent variables included: (1) characteristics of health facilities, including health facility preparedness, place of birth, place of hospitalization and its duration, KMC initiation, and maintenance support as reported by the mother; (2) characteristics of HCWs, including their knowledge, attitude, and skills on KMC implementation; (3) characteristics of the mothers, including age, education, employment status, number of children, knowledge, attitude, and KMC maintenance support at home; (4) characteristics of the babies, including sex, weight, and status at birth expressed as numbers and percentages.

KMC uptake as the outcome variable in the study included (1) day of life of KMC initiation and (2) KMC duration in hours on the day before discharge and seventh day after discharge of the small baby. Of the 227 babies recruited, 47.5% were hospitalized in only 1 facility, while the rest were hospitalized in 2 or more health facilities. Overall, 48.5% of the recruited babies were born and or hospitalized in the selected facilities of this study. Additionally, another 23.2% of small babies were born in a health facility that was excluded but were transferred to 1 of the selected health facilities, and 8.8% were born in 1 of the selected facilities but transferred to a health facility not chosen for this study. Thus, 80.2% of all the babies had access to any 1 of the selected health facilities for this study. Hence, to compute the bivariate association of KMC uptake with health facility preparedness, knowledge, attitude, and skills of HCWs, we used 1 or more of the following operations:

- Average scores obtained at the 2 time-point assessments of these variables were used because they were considered the best value to represent the period during which small babies were recruited.
- Average scores were computed if a baby was born in 1 health facility and transferred to another health facility.
- The mean score obtained for 3 PHCs selected for this study was allocated to any other PHC that was excluded, if the baby was born or hospitalized in such a facility, with the assumption that they were similar in characteristics.

Mothers’ occupations were varied; hence, occupation was categorized as employed and not employed. For outcome variables, the day of KMC initiation was categorized as 3 days or less and more than 3 days, and KMC duration on the day before discharge and the seventh day after discharge was categorized as less than 8 hours and 8 hours or more. Bivariate analysis was first performed to determine which variables could be included in multivariable analyses. These were expressed as unadjusted relative risk and 95% confidence intervals. Due to sample size, all variables selected for the log-binomial regression analysis could not be pushed to the analysis simultaneously. Thus, those variables with a *P* value ≤.10^40^ in bivariate analysis, plus those that were relevant based on the outcome assessed, were inserted into the model stepwise to perform the log-binomial regression analysis for identification of KMC uptake determinants. Hence, only those variables that were significantly associated in the log-binomial regression analysis are being reported here.

### Ethical Approval

Ethical approval was obtained from the St. Johns Medical College and Hospital Institutional Ethics Committee (Ref No 64/2017 on April 17, 2017) and the University of Stirling NHS, Invasive or Clinical Research Committee (Ref NICR 16/17, Paper 48 on May 25, 2017). Permission was obtained from the Government of Karnataka and administrative heads of public and private health facilities, respectively, for the conduct of the district-wide project within which this study was nested. All participants in the study provided their informed consent. Data were anonymized for analysis.

## RESULTS

The average health facility preparedness score was higher for private (n=1) compared to public health facilities (n=7), 65.0% versus 57.5%, respectively. Average scores on knowledge, attitude, and skills of all HCWs (n=79) were computed only for those who completed both time-point assessments. The average score of HCWs for knowledge (n=35), attitude (n=25), and skills (n=25) was 68.5%, 79.2%, and 57.5%, respectively.

The average health facility preparedness score was higher for private compared to public health facilities.

The mean (±standard deviation) age of mothers and fKMC providers was 23.5 (±4) and 36.9 (±13.9) years, respectively. Of the 209 mothers, 52.2% were unskilled workers, which included laborers and daily wage workers; 39.2% were unemployed (homemakers); and 8.6% were skilled workers. Nearly two-thirds (64.3%) of the spouses were unskilled workers, 28.0% were skilled workers, and 7.7% were engaged in business. More than two-thirds (64.1%) of mothers had an eighth grade or less education and 55% were first-time mothers (Table 1). Among the fKMC providers who responded to the questionnaire, 78.3% had an eighth grade or less education; 61.5% were unskilled workers, 31.3% were unemployed (homemakers or students), and 7.2% were skilled workers.

**TABLE 1.** Characteristics of Mothers and Babies Participating in KMC Uptake Study in Karnataka, India

**Variables**	**No. (%)**
Mothers (n=209)	
Education	
≤8^th^ grade	134 (64.1)
>8^th^ grade	75 (35.9)
Employed^a^	
Yes	127 (60.8)
No	82 (39.2)
Number of children	
1 child	114 (54.5)
>1 child	95 (45.4)
Babies <2,000 g at birth (n=227)	
Sex	
Male	94 (41.4)
Female	133 (58.6)
Birth weight	
≤1,500 g	48 (21.2)
>1,500 g	179 (78.9)
Place of birth^b^	
Public health facility	129 (56.8)
Private health facility	78 (34.4)
Home	20 (8.8)
Health status at birth^c^	
Well	37 (16.3)
Sick	190 (83.7)
Place of hospitalization^b^	
Public health facility	105 (46.3)
Private health facility	122 (53.7)
Hospitalization duration^b^	
≤3 days	102 (44.9)
>3 days	125 (55.1)

Abbreviation: KMC, kangaroo mother care.

^a^Those in skilled and unskilled occupation grouped as employed since only a small percentage were under skilled category for binomial and log binomial regression analysis.

^b^Were grouped under characteristics of health facilities for binomial and log binomial regression analysis.

^c^As reported in the district-wide project database.

The knowledge score of mothers was slightly above average, 57.7%, while their attitude score was 100%. The scores for mothers on KMC initiation and maintenance support at the health facility were 45.0% and 51.3%, respectively. Close to one-quarter (21.0%) of mothers had an fKMC provider at the health facility. At home, 47.4% of mothers had an fKMC provider. This included all but 2 of the mothers with twins. Support received from CHWs by mothers at home, a component of home KMC maintenance support, was 61.6%. Overall, KMC maintenance support at home was 44.5%.

Most of the small babies (78.9%) had a birth weight of more than 1,500 g, and 83.7% of them had a status of “sick” at birth (Table 1). Only 8.8% of the small babies were born at home, yet they were all hospitalized in a health facility after birth. Nearly half (44.9%) of all the small babies were hospitalized for 3 days or less.

Almost all (95.2%) small babies were initiated on KMC at the health facility. More than one-quarter (28.7%) of the babies were initiated on KMC on the first day of life, 30.9% on day 2–3 of life, 26% between day 4 and 7 of life, and 14.3% after the first week of life.

The median (interquartile range) duration of KMC provided to recruited babies increased from the day of its initiation (6.0 [2.0, 9.0]) to the day before discharge (8.0 [3.8, 11]) by 2 hours (n=216) and then decreased on the seventh day after discharge (6.0 [2.8, 10]) by 2 hours (n=219). Data were available for 71.4% of babies on the number of days KMC was provided. KMC was provided for 30.2±8.5 days with a range of 2 to 45 days. Almost three-quarters (71.6%) of mothers were continuing KMC on the day of the visit when the unadjusted age of the babies was 35.6±7.5 days (range 28–51 days or 4–7.2 weeks).

### Association of Independent Variables With Components of KMC Uptake

#### KMC Initiation

Babies hospitalized in a health facility with lower health facility preparedness were 2% more likely to be initiated early (3 days or less) on KMC (Table 2). Babies born and hospitalized in public health facilities were 374% and 383% more likely to be initiated early on KMC, respectively. Finally, babies hospitalized for 3 days or less were 321% more likely to be initiated on KMC early. Mothers who received more KMC initiation and maintenance support at the health facility were 6% and 4%, respectively, more likely to initiate KMC early. A more positive attitude and higher skills of HCWs were significantly associated with earlier KMC initiation. Babies weighing more than 1,500 g at birth were 56% times more likely to be initiated early on KMC.

**TABLE 2.** Bivariate Analyses of KMC Uptake by Characteristics of Health Facilities, Health Care Workers, Mothers, and Babies in Karnataka, India

Determinants	Day of Life of KMC Initiation, No. (%)(n=223)^a^^,^^c^		uRR(95% CI)	KMC Duration-Day Before Discharge,No. (%) (n=216)^a^^,^^d^		uRR(95% CI)	KMC Duration on 7^th^ Day After Discharge,No. (%) (n=219)^a^^,^^e^		uRR(95% CI)
	**≤3 days (n=133)**	**>3 days (n=90)**		**≥8 hrs (n=123)**	**<8 hrs (n=93)**		**≥8 hrs (n=98)**	**<8 hrs (n=121)**		
Characteristics of health facilities									
Health facility preparedness,^c^^,^^e^ mean±SD	61.1±14.6	65.1±6.5	0.98 (0.98, 0.99)	63.8±12.7	61.4±11.6	0.99 (0.98, 1.00)	61.0±13.2	64.0±11.2	0.98 (0.97, 0.99)
Place of birth^c^									
Public	94 (70.7)	33 (36.7)	4.74 (1.60, 13.5)	75 (60.9)	51 (51.6)	1.71 (0.84, 3.47)	56 (57.1)	69 (57.0)	1.10 (0.86, 1.40)
Private	22 (16.5)	54 (60.0)	1.73 (0.58, 5.12)	34 (27.6)	36 (41.9)	1.35 (0.66, 2.72)	29 (29.6)	45 (37.0)	0.63 (0.34, 1.17)
Home	17 (12.8)	3 (3.3)	1.0	14 (11.4)	6 (6.5)	1.0	13 (13.3)	7 (5.8)	1.0
Hospitalization location^c^^,^^d^^,^^e^									
Public	91 (68.4)	14 (15.6)	4.83 (2.91, 8.01)	76 (61.8)	28 (30.1)	2.15 (1.15, 3.07)	54 (55.1)	49 (40.5)	1.31 (1.01, 1.67)
Private	42 (31.6)	76 (84.4)	1.0	47 (38.2)	65 (69.9)	1.0	44 (44.9)	72 (59.5)	1.0
Hospitalization duration^c^									
≤3 days	87 (65.4)	15 (16.7)	4.21 (2.58, 6.86)	63 (51.2)	38 (40.9)	1.27 (0.93, 1.74)	48 (49.0)	53 (43.8)	0.91 (0.71, 1.16)
>3 days	46 (34.6)	75 (83.3)	1.0	60 (48.8)	55 (59.1)	1.0	50 (51.0)	68 (56.2)	1.0
KMC initiation support,^c^^,^^d^ mean±SD	6.8±1.6	5.8±2.7	1.06 (1.01, 1.11)	6.8±1.9	6.1±1.7	1.10 (1.04, 1.17)	6.5±2.1	6.03±2.1	1.03 (0.9, 1.11)
KMC maintenance support,^c^^,^^d^ mean±SD	8.5±3.5	7.0±3.9	1.04 (1.01, 1.07)	8.6±3.4	7.4±3.8	1.04 (1.003, 1.06)	8.0±3.9	8.1±3.6	0.99 (0.95, 1.03)
Characteristics of HCWs									
Knowledge,^e^ mean±SD	70.4±3.1	69.9±0.8	1.07 (0.98, 1.17)	70.2±2.7	70.3±2.3	0.99 (0.92, 1.06)	70.7±2.7	69.9±2.1	1.05 (1.01, 1.09)
Attitude,^c^^,^^d^^,^^e^ median (IQR)	77.0 (3.0)	71.0 (3.0)	1.09 (1.07, 1.11)	77.0 (6.0)	74.0 (5.0)	1.01 (0.99, 1.02)	76.0 (6.0)	74.0 (5.0)	1.02 (1.01, 1.05)
Skills,^c,d^ mean±SD	56.3±4.9	52.6±3.6	1.08 (1.05, 1.11)	55.8±5.0	53.7±4.2	1.05 (1.02, 1.08)	55.3±4.8	54.4±4.8	1.02 (0.99, 1.05)
Characteristics of mothers									
Age in years, mean±SD	23.3±3.6	23.9±4.5	1.03 (0.98, 1.07)	23.5±3.6	23.4±4.3	1.00 (0.97, 1.02)	23.5±4.5	23.6±3.5	0.99 (0.95, 1.03)
Education^d^									
≤8th grade	89 (66.9)	53 (58.9)	1.22 (0.89, 1.68)	85 (69.1)	52 (55.9)	1.37 (1.01, 1.84)	59 (60.2)	79 (65.3)	0.91 (0.70, 1.16)
>8th grade	44 (33.1)	37 (41.1)	1.0	38 (30.9)	41 (44.1)	1.0	39 (39.8)	42 (34.7)	1.0
Employed									
Yes	81 (60.9)	53 (58.9)	1.05 (0.76, 1.45)	78 (63.0)	51 (54.8)	1.22 (0.90, 1.65)	58 (59.2)	74 (61.2)	1.04 (0.81, 1.32)
No	52 (39.1)	37 (41.1)	1.0	45 (36.0)	42 (45.2)	1.0	40 (40.8)	47 (38.8	1.0
No. of children									
1 child	68 (51.2)	54 (60.0)	1.24 (0.89, 1.72)	69 (56.1)	50 (53.8)	1.05 (0.77, 1.43)	57 (58.2)	63 (52.1)	1.11 (0.88, 1.41)
>1 child	65 (48.9)	36 (40.0)	1.0	54 (43.9)	43 (46.2)	1.0	41 (41.8)	58 (49.9)	1.0
Knowledge,^b^ mean±SD		–	–	–	–	–	17.6±3.0	17.4±3.4	1.01 (0.96, 1.06)
Attitude,^b^ median (IQR)	–	–	–	–	–	–	4.0 (0)	4.0 (0)	–
KMC maintenance support at home,^b^ median (IQR)	–	–	–	–	–	–	12 (20.8)	17 (20.0)	0.99 (0.98, 1.01)
Characteristics of small babies									
Sex									
Male	53 (39.8)	39 (43.3)	0.91 (0.66, 1.26)	57 (46.3)	36 (38.7)	0.83 (0.60, 1.15)	43 (43.9)	48 (39.7)	0.92 (0.72, 1.18)
Female	80 (60.2)	51 (56.7)	1.0	66 (53.7)	57 (61.3)	1.0	55 (56.1)	73 (60.3)	1.0
Birth weight, g^c^									
≤1,500	18 (13.5)	29 (32.2)	0.56 (0.42, 0.76)	23 (18.7)	25 (26.9)	0.77 (0.56, 1.08)	23 (23.5)	26 (21.5)	0.95 (0.70, 1.27)
>1,500	105 (86.5)	61 (67.8)	1.0	100 (81.3)	68 (73.1)	1.0	75 (76.5)	95 (78.5)	1.0
Status at birth^c^^,^^d^									
Well	16 (12.0)	19 (21.1)	0.69 (0.48, 0.99)	13 (10.6)	21 (22.6)	0.64 (0.46, 0.88)	17 (17.3)	20 (16.5)	0.97 (0.7, 1.34)
Sick	117 (88.0)	71 (78.9)	1.0	110 (89.4)	72 (77.4)	1.0	81 (82.7)	101 (83.5)	1.0

Abbreviations: CI, confidence interval; HCW, health care worker; IQR, interquartile range; KMC, kangaroo mother care; SD, standard deviation; uRR, unadjusted relative risk.

^a^Subset of babies for whom KMC details were available in database.

^b^Not included in bivariate analysis for “day of life of KMC initiation” and “duration of KMC on day before discharge” since these were assessed for more than 28 days after childbirth.

^c^Variable selected for log binomial regression analysis for day of life of KMC initiation (*P* value up to .10).^40^

^d^Variable selected for log binomial regression analysis for KMC duration before discharge (*P* value up to .10).^40^

^e^Variables selected for log binomial regression analysis for day of life of KMC initiation for KMC duration on 7^th^ day after discharge (*P* value up to .10).^40^

#### KMC Duration on Day Before Discharge

Babies admitted to public health facilities were 115% more likely to receive 8 hours or more of KMC on the day before discharge. Better KMC initiation support increased the likelihood of 8 hours or more of KMC on the day before discharge by 10%, while better KMC maintenance support at the health facility increased this likelihood by 4%. A more positive attitude and higher skills of HCWs increased by 5% and 1%, respectively, the likelihood of 8 hours or more of KMC before discharge. Mothers with an education of eighth grade or less were 37% more likely to provide 8 hours or more of KMC before discharge, while babies who were sick at birth had a 36% higher likelihood of receiving 8 hours or more of KMC on the day before discharge.

#### KMC Duration on Seventh Day After Discharge

The babies who received 8 hours or more of KMC on the seventh day after discharge were predominantly from health facilities with lower health facility preparedness scores. Babies hospitalized in public health facilities had a 31% increased likelihood of receiving 8 hours or more of KMC on the seventh day after discharge compared to those in private health facilities. Higher knowledge of HCWs increased the likelihood of 8 hours or more of KMC on the seventh day after discharge by 5%, while a more positive attitude and higher skills scores of HCWs increased this likelihood by 2%.

Higher knowledge of HCWs increased the likelihood of 8 hours or more of KMC on the seventh day after discharge by 5%.

### Determinants of KMC Uptake at the Health Facility

#### Day of Life of KMC Initiation

Being hospitalized in a public compared to a private health facility increased early KMC initiation by 168% (*P*=.007) (Table 3). A more positive attitude of HCWs facilitated earlier KMC initiation by 1% (*P*=.042). Better KMC initiation support at the health facility received by the mothers improved early KMC initiation by 3% (*P*=.045).

**TABLE 3.** Log Binomial Regression Analysis of Determinants of KMC Uptake in Karnataka, India

Determinants^a^	Referent Group^b^	Adjusted RR (95% CI)	*P* Value
KMC initiation (≤3 vs. >3 days)			
Place of hospitalization	(Public vs. private^b^)	2.68 (1.31, 5.51)	.007
HCWs’ attitude	(Median 77 vs. 71^b^)	1.01 (1.00, 1.01)	.042
KMC initiation support at health facility	(Mean 8.5 vs. 7.0^b^)	1.03 (1.02, 1.04)	.045
KMC duration on day before discharge from health facility (≥8 vs. <8 hrs)			
HCWs’ skills	(Mean 55.8 vs. 53.7^b^)	1.05 (1.01, 1.07)	.017
KMC maintenance support at facility	(Mean 8.6 vs. 7.4^b^)	1.03 (1.003, 1.06)	.023
KMC duration on 7^th^ day after discharge from health facility (≥8 vs. 8 hrs)			
Place of hospitalization	(Public vs. private^b^)	1.31 (1.02, 1.68)	.035
HCWs’ knowledge	(Mean 70.7 vs. 69.9^b^)	1.02 (1.01, 1.04)	.039

Abbreviations: CI, confidence interval; HCW, health care worker; KMC, kangaroo mother care; RR, relative risk.

^a^Due to the small sample size, all variables selected could not be pushed to the log-binomial regression analysis model simultaneously. Instead, variables were inserted into the model stepwise. Hence, only those variables that are significantly associated are reported.

^b^Referent group.

#### KMC Duration on Day Before Discharge

Duration of 8 hours or more of KMC improved by 5% with higher skills of HCWs (*P*=.017) after other covariates were adjusted. Better KMC maintenance support at the health facility received by the mothers increased the likelihood of 8 hours or more of KMC on the day before discharge by 3% (*P*=.003).

### Determinants of KMC Uptake at Home

#### KMC Duration on Seventh Day After Discharge

Hospitalization in public health facilities and higher knowledge of HCWs increased KMC duration of 8 hours or more on the seventh day after discharge by 31% (*P*=.035) and 2% (*P*=.039), respectively.

Thus, the hypothesis that (1) health facility preparedness increases early initiation and duration of KMC before discharge was rejected; (2) HCWs’ optimal knowledge, attitude, as well as skills are likely to impact uptake of KMC by mothers was accepted; and (3) mothers who are supported at the health facility by HCWs and at home by family members and CHWs are likely to practice KMC for longer duration at the health facility and subsequently at home, respectively, was accepted partly because only support received at the health facility impacted duration of KMC.

## DISCUSSION

This study reports the determinants of KMC initiation and maintenance along the health facility to community continuum for those stable small babies who survived 4–8 weeks of unadjusted age within the subdistrict.

The public health facilities fared better in facilitating early KMC initiation than private health facilities.

### Early Initiation of KMC

Early and immediate KMC initiation and its maintenance are known to improve health status and impact survival of stable and sick small babies.^12^^,^^18^^,^^19^ The small babies recruited in this study could be considered physiologically stable because none of them required intensive therapy, yet only 59.6% were initiated on KMC early (3 days or less of life). Characteristically, the public health facilities—PHCs, CHCs, and subdistrict hospital—fared better in facilitating early KMC initiation than private health facilities. We observed that the primary-level public health facilities (CHCs and PHCs) had a lower health facility preparedness score compared to the private health facility for lack of specialists and support staff. Yet probably because these health facilities had 4–30 adult inpatient beds—unlike the private health facilities that offered neonatal services to 10–20 babies—and not a very high inpatient load, HCWs of public health facilities were able to facilitate early initiation of KMC for small babies. Nonetheless, assuming physiologically stable babies were more often hospitalized in public than private health facilities, their turnover would be more frequent, supporting early KMC initiation at public health facilities. None of the public health facilities in this subdistrict had a special newborn care unit or newborn unit nor specialist doctors to care for small babies during the study period. Previous studies had cited the fear of nosocomial infections, health workforce shortage and lack of space, lack of time for counseling mothers, and lack of training as reasons for the delay in KMC initiation.^21^^–^^23^ Other possible reasons could be poor coordination with the government and competing business interests.^30^^,^^41^ Reasons for the delay in KMC initiation at private health facilities were not explored in this study, but the above reasons could be plausible in this setting. Hence, in a setting where the private sector plays a substantial role in provision of neonatal services, it will be vital for public health officials to engage with private sector facilities to ensure that they also follow standard recommendations for ENC of small babies to reach the expected targets of more than 80% coverage of small babies with KMC.^18^

To reach KMC coverage targets, it will be vital for public health officials to engage with private sector facilities to ensure they follow standard recommendations for ENC of small babies.

Findings from previous studies highlighted the importance of health facility preparedness for KMC implementation.^21^^–^^24^ Yet, this study did not show any association between health facility preparedness and early KMC initiation, probably because the district-wide project advocated preparedness of all health facilities for KMC implementation. Another possible reason for this finding could be methodological constraints. The tool used to measure health facility preparedness in this study was investigator-developed, unlike the tool used by Bergh et al.^42^ This study included primary and secondary public and private health facilities, while Bergh et al. measured preparedness of district health facilities for KMC practice. The tool only identified the availability of trained and specialist health workforce; documentation and reporting of KMC; designated spaces with amenities such as food, water, and adjustable beds for mothers; relevant education materials, devices, and equipment for comfortable KMC practice; and a policy on KMC. Yet, no direct observation of KMC uptake was made, as this was measured through quality control methods in the district-wide project.^29^^–^^30^

Although minimal, KMC initiation support at the health facility received by mothers significantly increased earlier KMC initiation, indicating it was sufficient and valuable^43^^–^^44^ to facilitate KMC uptake. KMC was not part of ENC of small babies in this district prior to the district-wide implementation research project.^29^ With time and continued support mechanisms for HCWs, it is possible that health facilities there would improve their capabilities to support mothers for earlier KMC initiation. A more positive attitude of HCWs facilitated earlier KMC initiation in this study through counseling and assisting mothers to initiate KMC—denoting that HCWs acknowledged the significance of early KMC initiation on morbidity and mortality of small babies. More than 80% of the babies recruited in this study were hospitalized in the selected health facilities. There may have been bias in the purposive sampling of HCWs. However, mothers were able to recall the support they received through HCWs even 1 month after the birth of the small baby. Although support received was scored as minimal, it still was a determinant of early KMC initiation. Even though other factors, such as the presence of amenities for the mother and the ambience of the environment, are known to have an impact on KMC uptake,^21^^–^^24^^,^^36^ this was not confirmed by the findings of this study.

Given that 2-way referrals between private and public health facilities are a common occurrence in this subdistrict, strategic networking for follow-up needs to be considered to facilitate KMC maintenance once initiated. CHWs who are incentivized to accompany mothers for childbirth to public health facilities or even during referral could be an ideal resource to create these networks. These facts, and possibly the Hawthorne effect^30^ of the presence of external district-wide project staff, could have resulted in early KMC initiation. One must be mindful that 10% of small babies recruited were discharged by day 2 of life, which has important lessons for KMC implementation along the health facility to community continuum. Knowledge and proficiency in correct positioning, monitoring, and providing sufficient daily KMC duration safely for small babies are required of mothers, especially those with twins, before discharge from the health facility. HCWs need to ensure these requirements are met before discharge and that responsibility to follow up is transferred to CHWs smoothly so that KMC is continued at home for 4–6 weeks of life. Thus, the social context and health facility to community dynamics are important considerations for KMC continuation once initiated.^21^^,^^25^^,^^26^^,^^36^ Early initiation, as early as soon after birth, of a stable small baby at the health facility would provide the mother an opportunity for supervised KMC practice in a supportive environment. The sociodemographic profile of the mothers, in terms of their education and occupation, of this cohort of small babies could be similar to that of mothers from other LMICs. Hence, these findings could likely be applied to mothers from such settings where education and income levels are below average and early discharge is a phenomenon. Such mothers would probably require small capsules of information, assistance, and supervision of KMC to enhance their competence in its provision during the shortened hospitalization period.

#### KMC Duration at the Health Facility

Daily duration of SSC—a component of KMC for 8 hours or more, especially in the first 2 days of life and continued until required—has a known impact on morbidity and mortality of LBW babies.^18^^–^^20^ As the first point of contact for these vulnerable babies in rural and remote areas, HCWs, especially nurses, need to play a significant role at the start of life to ensure SSC as early as possible. With the current health policy in support of KMC, it is crucial that HCWs receive support to gain appropriate knowledge, attitude, and skills to implement KMC for small babies.^2^^,^^39^^,^^41^ The skill of HCWs and the KMC maintenance support mothers received at the health facility were significantly associated with KMC duration on the day before discharge from the health facility. Resources are available at the health facility to support the mother for provision of KMC. These could primarily include the nurse and health assistants, along with the doctor, counselors, and peer mothers at the health facility.^30^^,^^43^ KMC daily duration could be enhanced further with collaborative support from all these resources.^23^ The presence of an fKMC provider could also improve KMC duration at the health facility.^23^^,^^24^^,^^26^ HCWs could take the cue to identify, educate, and motivate 1 or 2 family members on KMC. These family members could then become fKMC providers of supervised SSC at the health facility so that they gain confidence to continue SSC at home.

With the current health policy in support of KMC, it is crucial that HCWs receive support to gain appropriate knowledge, attitude, and skills to implement KMC for small babies.

HCWs who have a positive attitude and are skilled^23^^–^^24^ are known to influence KMC uptake. Yet, although this study demonstrated a significant association of HCWs’ attitudes with initiation of KMC, the duration of KMC before discharge was not associated with attitude. This finding could be due to the sample size. One could extrapolate that the skills of HCWs could have facilitated the mothers to learn this new behavior of providing KMC within a short span of 1–3 days following childbirth and that mothers are more than willing to support the baby in any way possible. Further, the KMC maintenance support provided through the KMC kit and presence of an fKMC provider and others to assist her at the health facility suggests that they were comfortable enough to provide KMC for longer duration with ease, despite their babies being small. The provision of the KMC kit to secure the baby safely could have further increased their confidence to position the baby correctly and continue KMC.^44^ However, one must view these findings with caution because mothers may have provided the recommended duration of SSC for early discharge. Consequently, mothers, spouses, and other family members must be reminded that SSC and exclusive breastfeeding need to be continued with the same rigor at home following discharge from the health facility.

#### KMC Duration at Home

Better knowledge of HCWs was associated with the provision of 8 hours or more of KMC on the seventh day after discharge from the health facility (Table 3). Findings from this study show that HCWs’ attitude and KMC initiation support was associated with early initiation of KMC, their skills and KMC maintenance support were associated with KMC duration before discharge, and now their knowledge associated with KMC duration at home. This indicates that the overall competence of HCWs is necessary for them to facilitate KMC uptake along the health facility to community continuum.^29^^–^^30^ Similarly, a previous study reported that information and practical advice provided by HCWs on assistance and encouragement in positioning the babies for KMC was valuable for mothers to continue KMC.^45^ Despite most of the mothers in this setting being from homes with limited amenities, often with a single bed for more than 5 family members, they were still able to continue KMC at home, pointing indirectly to their awareness of the benefits of KMC and the support^26^^,^^27^^,^^43^ they received at home through fKMC providers or family members for domestic chores. Their knowledge of and the possibility of experiencing the benefits of KMC could have motivated them to sustain KMC uptake even at home. Nevertheless, KMC maintenance support at home after discharge from the health facility was not significantly associated with KMC duration at home. KMC maintenance support at home included help from family members for domestic chores; support from fKMC providers for provision of SSC, including the knowledge, attitude, and support they received for KMC uptake; and support from CHWs. Methodologically, the computation of KMC maintenance support at home could have been a limitation and a probable reason for the above finding. Further exploration to quantify the association of duration of KMC at home or in the community with support received independently from family members, fKMC providers, and CHWs is required.

The findings indicate that the overall competence of HCWs is necessary for them to facilitate KMC uptake along the health facility to community continuum.

Babies who were hospitalized in public over private health facilities continued to receive 8 hours or more of KMC on the seventh day after discharge in this study. The probability that HCWs in public health facilities are better connected with CHWs^21^^,^^22^^,^^28^ could have placed these facilities at an advantage over private facilities, resulting in this finding.^30^ The study showed that most of the KMC hours and days provided to small babies occurred after discharge. The availability of competent CHWs to support mothers at home^29^^–^^30^ could have complemented this finding along with the presence of fKMC providers, indicating from this study that fKMC providers, other family members^21^^,^^25^^,^^43^ and CHWs are key stakeholders in ensuring that KMC uptake is sustained at home until required, although this finding would need further exploration.

Neither the characteristics of mothers—such as their knowledge and attitude on KMC (although cited in literature as being important for KMC uptake^21^^,^^25^^,^^43^) or their education, occupation, number of children, and age—nor the characteristics of small babies (weight, health status at birth, or sex) were associated with KMC uptake. Yet, the fact that mothers, irrespective of these variables, continued KMC for more than 3–8 hours and for longer than 28 days at home signifies that they adopted KMC as an essential intervention to improve the health and well-being of their babies.

### Recommendations

The key determinants associated with KMC initiation and maintenance uptake along the health facility to community continuum in a predominantly rural subdistrict of northern Karnataka were evident through this study. These determinants are relevant globally for improving KMC uptake. HCWs and CHWs from similar settings could perhaps benefit from this study to make appropriate recommendations on early KMC initiation and its maintenance both at the health facility and home. Any government policy initiative should consider the key role that private health facilities play in filling the gap of vital services for small babies unavailable at public health facilities in this setting and other similar settings globally. Strategies to build capacity and incentivize private health facilities either monetarily or through recognition of their role in care of small babies would be essential to get their buy-in for facilitating early initiation of KMC. Efforts of the health facility leadership to build on the competence of the health workforce would be crucial for them to support mothers for KMC uptake along the continuum. The competence of HCWs in KMC implementation is indirectly relevant to their ability to support mothers. Their skills, attitude, and knowledge were central for its uptake and were associated with increased KMC duration both at the health facility and after discharge from the health facility. Based on these findings and their implications, we propose a framework for furthering KMC uptake along the health facility to community continuum (Table 4).

**TABLE 4.** Proposed Operational Framework for Catalyzing KMC Uptake Along the Health Facility to Community Continuum Based on Study Findings in Karnataka, India

**Community**	**Health Facility**	**Community**
Antenatal care	Pregnancy + childbirth + hospitalization of the low birth weight baby	Continuation of KMC for 4–6 weeks at home
**CHWs’ Role** ^a^	**HCWs’ Role**	**CHWs’ Role**
Educate and counsel mothers and the community on KMC and exclusive breastfeeding Inform about need for childbirth at a health facility Identify peer mothers as KMC champions Identify potential foster KMC providers	Support mothers at antenatal visits on KMC Ensure support from all HCWs, including peer mothers and foster KMC providers, for mothers to initiate and maintain KMC Recognize HCWs who promote KMC as champions Ensure foster KMC providers are identified and that they begin supervised KMC provision at the health facility before discharge	Reinforce information on KMC through home visits after discharge daily for a week; find ways to enhance KMC duration Encourage foster KMC providers to provide skin-to-skin contact or assist with household chores Ensure mothers monitor babies during KMC

Abbreviations: CHW, community health worker; HCW, health care worker; KMC, kangaroo mother care.

^a^Extrapolated from this study’s findings.

Community mobilization and education of pregnant mothers and family members on KMC, although not explored in this study, could be vital given the increasing proportion of women having childbirth at health facilities, with early discharge of the stable small baby.^20^ This would help them to better assimilate information on KMC after childbirth in the event of the birth of a small baby. Unequivocally, from the mother’s perspective, it is vital to receive support at the health facility for (1) KMC initiation through provision of information and counseling and (2) its maintenance by HCWs who are competent with KMC implementation across all types of public and private health facilities (primary and secondary). Mothers would also require support from CHWs and family members at home to continue KMC for as long as required.^30^ This study occurred while the district-wide study was being conducted; hence, project team members received feedback on ways that HCWs and CHWs could be supported based on information provided by mothers.

Programmatic priority must be given to enhancing the competence of CHWs to ensure KMC initiated at the health facility is continued at home after discharge, in the context of early discharge of stable small babies and the role of private health facilities in care of small babies. Skill-based workshops and support mechanisms can yield systematic improvements in the competence of both HCWs and CHWs with a focus on improving their ability to counsel, educate, and assist mothers inclusive of family members for KMC uptake.^30^^,^^45^^,^^46^ The shortened hospitalization necessitates that CHWs are motivated to make daily home visits to encourage both mothers and family members for the first week after discharge to sustain KMC duration, which seemed to dip a week after discharge. CHWs will also need communication and advocacy skills to enable family members to support mothers both in provision of SSC and domestic chores.

Programmatic priority must be given to enhancing the competence of CHWs to ensure KMC initiated at the health facility is continued at home after discharge.

## CONCLUSION

This study demonstrated that mothers and family members are willing to provide KMC for their small babies. Competent, supportive HCWs are key to early initiation and longer duration of KMC before discharge and on the seventh day after discharge from the health facility. These findings could be universally applied in any similar sociodemographic setting, given the benefits of KMC uptake on the health and well-being of small babies and mothers. Thus, efforts of health facility managers must be focused on building KMC competencies (knowledge, attitude, and skills) for both HCWs and CHWs through supportive mechanisms to facilitate sustainable KMC uptake along the health facility to community continuum.

## Notes

**Peer Reviewed**

**First published online:** May 25, 2023.
